# Impact of Hepatitis C Virus (HCV) infection on biomolecular markers influencing the pathogenesis of bladder cancer

**DOI:** 10.1186/1750-9378-8-24

**Published:** 2013-06-28

**Authors:** Kamel Z Hemmaid, Amira Awadalla, Essam Elsawy, Abdel-aziz M Hussein, Azza Abdel-aziz, Ahmed A Shokeir, Ahmed S El-Hefnawy, Hassan Abol-enein

**Affiliations:** 1 Department of Zoology, Faculty of Science, Zagazig University, Zagazig, Egypt; 2 Urology and Nephrology Center, Mansoura University, Mansoura, Egypt; 3 Department of Physiology, Faculty of Medicine, Mansoura University, Mansoura, Egypt; 4 Department of Pathology, Faculty of Medicine, Mansoura University, Mansoura, Egypt

**Keywords:** HCV, Bladder cancer, Telomerase, Retinoblastoma gene, E2F3, TP53, CDKN1A (p21), FGFR3

## Abstract

**Objective:**

The present study was designed to determine the possible impact of hepatitis C virus (HCV) infection on the expression of telomerase (TERT), retinoblastoma (RB1), E2F3, TP53, CDKN1A (p21) and fibroblast growth factor receptor- 3 (FGFR3) genes in patients with bladder cancer (BC).

**Materials and methods:**

100 patients with bladder cancer (15 female and 85 male) were divided into 2 groups; Group I: 50 HCV negative subjects (age range 36–79), and Group II: 50 HCV positive subjects (age range 42–80). Expressions of the telomerase, retinoblastoma (Rb), E2F3, TP53 and FGFR3 genes were tested by immunohistochemistry and real time PCR in tumour tissues and healthy bladder tissues. Also, telomerase activity was assessed by telomeric repeats amplification protocol (TRAP).

**Results:**

Bladder tumors associated with HCV infection were of high grade and invasive squamous cell carcinomas (SCCs). Expressions of hTERT, Rb, E2F3, TP53 and FGFR3 as well as telomerase activity were significantly higher in bladder tissues of HCV-infected patients compared with bladder tissues of non infected patients (p<0.05). On the contrary, CDKN1A (p21) expression was significantly lower in bladder tissues of HCV-infected patients compared to bladder tissues of non infected patients (p<0.05).

**Conclusion:**

The expressions of hTERT, Rb, E2F3, TP53 and FGFR3 as well as the activity of telomerase were significantly high in malignant bladder tissues associated with HCV infection. On the other hand, CDKN1A (p21) expression was low in bladder tissues of HCV-infected subjects. Moreover, there was a positive correlation between HCV infection and expression of telomerase, E2F3, TP53 and FGFR3. There was a negative correlation between HCV infection and expression of Rb and p21.

## Background

Bladder cancer (BC) in Egypt is the most prevalent cancer in men (16%) and is the second most common cancer in women, producing >7900 deaths annually, which is strikingly higher than most other parts of the world [1]. The pathogenesis of bladder cancer is a complex process that involves the activation of proto-oncogenes [2], inactivation or loss of tumor suppressor genes [3] and mutations of cell cycle regulatory genes [4]. Tumour suppressor genes involved in development of BC include retinoblastoma (Rb) and TP53 genes. Rb gene mutations are seen in approximately 30% of BC [5]. A recent study reported that Rb gene removal and over expression of E2F3 may be required for bladder carcinogenesis [6] as well as overexpression of TP53 [7]. Fibroblast growth factor receptor-3 (FGFR3) gene is a proto-oncogene that promotes cell survival [8] and mutations of the FGFR3 gene are associated with early papillary lesions with low malignant potential [9,10]. These mutations have been found in 75% of non-dysplastic genuine urothelial papillomas [10], indicating that they are very early events in the papillary tumor development. Moreover, CDKN1A (p21) functions as a regulator of cell cycle progression at G_1_ phase and altered expression was demonstrated in more than half of pT1 bladder tumors [7].

Telomerase is a specialized ribonucleoprotein complex including an RNA component, human telomerase RNA (hTR), and a catalytic protein, telomerase reverse transcriptase (hTERT), which stabilizes the telomeres of linear chromosomes [11,12]. Expression of hTERT mRNA is very closely associated with telomerase activity in human tumors and can be detected by reverse transcription polymerase chain reaction (RT-PCR) [13] and immunohistochemical (IHC) methods [14]. Most human tumors display high levels of telomerase activity [13,15]. Such expression in cancer cells might be a necessary step for tumor initial development, progression [15] and plays an important role for long-term maintenance [11,16]. Recently, *Shaker et al.*[17] concluded that telomerase may be involved in the pathogenesis of schistosomal BC and demonstrated an increase in telomerase activity assessed by the telomeric repeats amplification protocol (TRAP).

The role of hepatitis C virus (HCV) as an etiologic agent of hepatocellular carcinoma (HCC) has been established [18,19]. Also, there is a relationship between HCV infection and other tumors such as oral squamous cell carcinoma [20]. In a recent study, Gordon *et al.* was found that the risk for renal cell carcinoma becomes nearly double in patients with chronic hepatitis C infection [21]. In Egypt, in the past, the incidence of schistosomiasis infection was high, but nowadays, HCV infection predominates with approximately 10% to 20% of the population being infected [22]. The frequency of histological cell type of bladder carcinoma was significantly changed over the past two decades [23]. In a past report, squamous cell carcinoma (SCC) predominated (59%) over transitional cell carcinoma (TCC) (31%). Now, the relative frequency is 64% for TCC, 29% for SSC, 5% for adenocarcinoma and 2% for undifferentiated carcinoma. We hypothesized the change in histopathological profile of BC could be due to the change in the risk factor. To test this hypothesis, we investigated in the current study, the expression of telomerase (TERT), some tumour suppressor gene (Rb, E2F3, TP53 and CDKN1A (p21)) as well as some proto-oncogenes (FGFR3) that influence pathogenesis of BC in HCV infected patients and compare their expressions with those in the non-HCV infected patients. Expressions of these genes were correlated with the clinic-pathological features of BC.

### Subjects and methods

#### Subjects and study design

One hundred subject with bladder cancer (15 female and 85 male) admitted to the Urology and Nephrology Center at Mansoura University during the period from Jan 2009 to Jan 2012 were enrolled in this study. They were divided into 2 main groups: Group I: 50 HCV negative subjects (age range 36–79). Group II: 50 HCV positive subjects (age range 42–80). In each group, 2 samples were taken from each enrolled subject, one from the malignant tissue and the other from the healthy surrounding urothelium. Tumor specimens and healthy urothelium were taken by cystoscopy (transurethral resection biopsies, TURB) and cystectomy. Patients were subjected to full clinical examination (general and abdominal examination), digital rectal examination (DRE), bimanual examination under anesthesia, routine laboratory investigations (liver function tests; albumin, bilirubin, and enzymes and kidney functions test; serum creatinine and blood urea nitrogen (BUN)), complete urine analysis, abdominal and pelvic ultrasonography, plain x-ray of the urinary tract, intravenous urography (IVU), and cystoscopy.

The study protocol was approved by the Ethical Committee of TBRI according to the Institutional Committee for the Protection of Human Subjects and adopted by the 18^th^ World Medical Assembly, Helsinki, Finland. Informed consents from all patients who underwent cystoscopy and biopsy from apparent growth and lesions were taken.

Patients with history of intravesical or systemic chemotherapy or immunotherapy -like mitomycin C or Bacillus Calmette–Guérin (BCG) vaccine, radiotherapy, urinary bilhariaziasis or smoking were excluded from this study. Also, immunocompromized patients or those having chronic cystitis were not included in this study.

### Routine histopathology

Each specimen of bladder biopsy from malignant tissues and normal urothelium was divided into two parts: a small fresh part was frozen for PCR and the large portion was fixed in 10% buffered formalin. The paraffin blocks were retrieved and 3 μm thickness sections were prepared for routine H&E. Other sections were prepared on charged slides for immunohistochemistry. Examination of slides from each specimen was done using Olympus CX51 light microscope. Pictures were obtained by a PC-driven digital camera (Olympus E-620). The computer software (Cell*, Olympus Soft Imaging Solution GmbH) enabled analysis to be performed. In all cases a histopathological diagnosis was made according to the World Health Organization histological classification of urothelial tumors [24].

### Immunohistochemical examination of hTERT, Rb, p53, FGFR3 and CDKN1A (p21)

Deparaffinized sections from all specimens were incubated for 30 min with 0.3% hydrogen peroxide in methanol and microwave heated in citrate buffer (pH 6.0) for 20 minutes. Subsequently, an indirect immunoperoxidase technique was applied, using monoclonal antibodies for *Telomerase (hTERT)* (clone 2C4, concentrated with 1:500 used dilution; Abcam catalogue No ab5181), *Retinoblastoma (Rb)* (clone 1F8, prediluted; Thermo scientific, catalogue No #MS-107-R7), TP53 (monoclonal mouse anti-human antibody DO-7; Dako, Carpinteria, CA; dilution 1:4000), CDKN1A p21 (monoclonal mouse anti-human, SX118, Dako; dilution 1:2000, FGFR3 (monoclonal mouse anti-human, clone B-9; Santa Cruz Biotechnology). Immunostaining was performed using Immuno-Pure Ultra-Sensitive ABC Peroxidase (Thermo Scientific Cat. No #32052) with (DAB) as chromogen. Proper positive and negative controls were performed. Breast carcinoma was used as positive control for Rb and TP53, pancreatic carcinoma for telomerase and colorectal carcinoma for CDKN1A (p21). As a negative control, sections were stained without the addition of a primary antibody.

### Interpretation of immunohistochemical staining

As for the immunohistochemistry assessment, slides were scanned by X40 magnification. Ten cellular areas were selected (i.e. the so-called hot spots) and evaluated at X400 magnification. Telomerase immunostaining was considered positive if at least 5% of the suspected cell population showed positive intranuclear dot-like telomerase staining. Quantitative assessment was done according to the method of Yan *et al.*[25]. Labeled cells were expressed as a percentage of tumor cells with positively stained nuclei divided by the total number of tumor cell nuclei counted. As for Rb expression, tumors were placed in one of two categories, altered or normal. Tumors with normal expression showed nuclear heterogeneous staining (less than 50%). Tumors with no Rb expression and those with a strong homogeneous staining pattern (more than 50%) were categorized as having altered Rb status [26]. TP53 was considered positive when samples demonstrated at least 10% nuclear immunoreactivity [27]. CDKN1A (p21) was considered altered when samples demonstrated no detectable or only very low levels of CDKN1A (p21) nuclear staining [26]. Expression of FGFR3 was evident as membranous and cytoplasmic immunoreactivity, scored in a semi-quantitaive scoring system: 0 = all tumor cells negative, 1 = weak positivity in more than 10% of tumor cells, 2 = moderate positivity and 3 = strong positivity/overexpression [27].

### Real time PCR for studied genes

#### RNA extraction and cDNA synthesis

According to the manufacturer’s instructions, total RNA from frozen tumor and from the corresponding non-cancerous tissue specimens were isolated by disruption of 50–100 mg tissues in 1 ml of TRIzol (Invitrogen Corporation, Grand Island, NY, USA). RNA was quantified spectrophotometrically and its quality was determined by agarose gel electrophoresis and ethidium bromide staining. Only samples that were not degraded and showed clear 18 S and 28 S bands under ultraviolet light were used for real-time PCR (RT-PCR). Reverse transcription was performed using 1 μg total RNA and the high capacity cDNA archive kit.

### Primers and probes

The primers and probes were purchased from Applied Biosystems. All the primers and probes of the studied genes are listed in Table 1.

**Table 1:** List of primers and probes used for the respective genes

**Probe**	**Reverse primer**	**Forward primer**	**Genes**
5'FAM-CAGCACTTCTTTTGAGCACACGGTCG-3'TAMRA	CAGTGGTTTAGGAGGGTTGCTT	TTCCAGAAAATAAATCAGATGGTATGTAA	**Rb1**
5'FAM- TTGCAAAGCATTGGAATCAGACAGCACT- 3'TAMRA	CTCGGCCCTCTTTTCTCTG	GCACTGGCTGATGAGTGTGT	**hTERT**
	AGCACCGCCGTCTGGTTGGC	CGGCAGTGGCGGTGGTGGTG	**FGFR3**
5'FAM-CTGTGACTTGCACGTACTCCCCTGCC- -3'TAMRA	AAGACCTGCCCTGTGCAGC	CGTCTGGGCTTCTTGCATTC	**TP53**
5'FAM-CGGCAGACCAGCATGACAGATTTCTACCA-3'TAMRA	GAGGAAGCCTAATCCGCC	CTGGAGACTCTCAGGGTCGAAA	**CDKN1A (P21)**
5'FAM-CAAGCTTCCCGTTCTCAGCC- 3'TAMRA	GAAGATGGTGATGGGATTTC	GAAGGTGAAGGTCGTAGTC	**GAPDH**

### Real time PCR reaction

The reaction was performed using a total volume of 50 μl containing 25 μl 1× TaqMan® Universal PCR Master Mix, 2.5 μl 20X TaqMan® Gene Expression Assay Mix (which BRAND?) and 22.5 μl of cDNA diluted in RNase-free water. The cycling parameters were as follows: initial denaturation at 95°C for 10 minutes, followed by 40 cycles consisting of denaturation at 95°C for 15 seconds, annealing at 60°C for 1 minute, extension at 72°C for 1 minute. Data analysis was carried out using ABI prism 7000 by equation 2-^ΔΔ^ ct [28].

### Assessment of telomerase activity using telomerase repeat amplification protocol (TRAP)

Five sections from each paraffin embedded tissue specimens were deparafinized by xylene, homogenized with homogenizer in 200 μl of cold lysis buffer. After 30 minute of incubation on ice, the lysates were centrifuged at 10,000 ×g for 30 min at 4°C. All steps were performed according to a previously described technique [17]. The assay kit (Telo-TAGGG Telomerase PCR ELISA plus) was supplied by Roche (Roche Diagnostics, Mannheim, Germany). The absorbance at 450 nm was determined. To confirm product specificity, a negative control was performed for each sample by heat inactivation of telomerase at 85°C for 10 min. The relative telomerase activity (RTA) in each sample was determined in relation to IS and the control (provided within the kit) readings using the formula provided by the manufacturers.

### Statistical analysis

All statistical analyses were done using Statistical Program for Social Sciences (SPSS 16.0). Qualitative data were presented as numbers and percents, while quantitative data were expressed as means ± SD. Chi-square test of independence was used for evaluating the significant association of histopathology type, tumour grade, tumour invasiveness, staging, and immunohistochemical staining of tumour markers with HCV infected and non-HCV patients. Pearson´s correlation was used to measure the relation between 2 variables. A significant correlation between two variables was taken at the 95% confidence interval. Comparison between means of different groups was done using one way ANOVA with Scheffe’s posthoc test.

## Results

### The clinicopathological features of bladder cancer in HCV-infected and non-HCV infected patients

Bladder tumors associated with HCV infection were of the transitional cell carcinoma (TCC) rather than squamous cell carcinoma (SCC), high grade rather than low grade and invasive rather than non-invasive. On the other hand, there was no association between HCV-associated tumors and LN affection, metastases and tumour staging. Also, there was no significant association between age and sex and HCV-associated tumours. Table 2 shows that HCV-associated tumors occur in early age (minimum age was 36 years); while non-HCV-associated tumours occur in late age (minimum age was 42 years).

**Table 2:** Clinicopathological features of bladder cancers in HCV-infected patients and non-infected patients

**Criteria**	**HCV infected patients**	**Non-HCV patients**	**P value**
**Age**	57.6±8.59	57.9±9.45	NS
	Minimum = 36	Minimum = 42	
	Maximum = 79	Maximum = 80	
**Sex**			
- Male	43 (86%)	42 (84%)	NS
- Female	7 (14%)	8 (16%)	
**Histopathological type**			
- TCC	32 (64%)	19 (38%)	p= 0.009
- SCC	18 (36%)	31 (62%)	
**Tumour grade**			
- Grade I	10 (20%)	26 (52%)	p= 0.000
- Grade II	19 (38%)	19 (38%)	
- Grade III	21 (42%)	5 (10%)	
**Lymph Nodes (LNs)**			
- Negative LNs	38 (76%)	40 (80%)	NS
- Positive LNs	12 (24 %)	10 (20%)	
**Tumour staging**			
**-** Stage (Ta)	1 (2%)	5 (10%)	p= 0.002
**-** Stage (T1)	12 (24%)	17 (34%)	
**-** Stage (T2)	13 (26%)	15 (30%)	
**-** Stage (T3)	12 (24%)	8 (16%)	
**-** Stage (T4)	12 (24%)	5 (10%)	
**Metastases**			
- Negative	43 (86%)	42 (84%)	NS
- Positive	7 (14%)	8 (16%)	

P= chi square test with significance of p ≤ 0.05.

### Immunohistochemical localization of hTERT, Rb, TP53, CDKN1A (P21) and FGFR3

**Figure 1:**
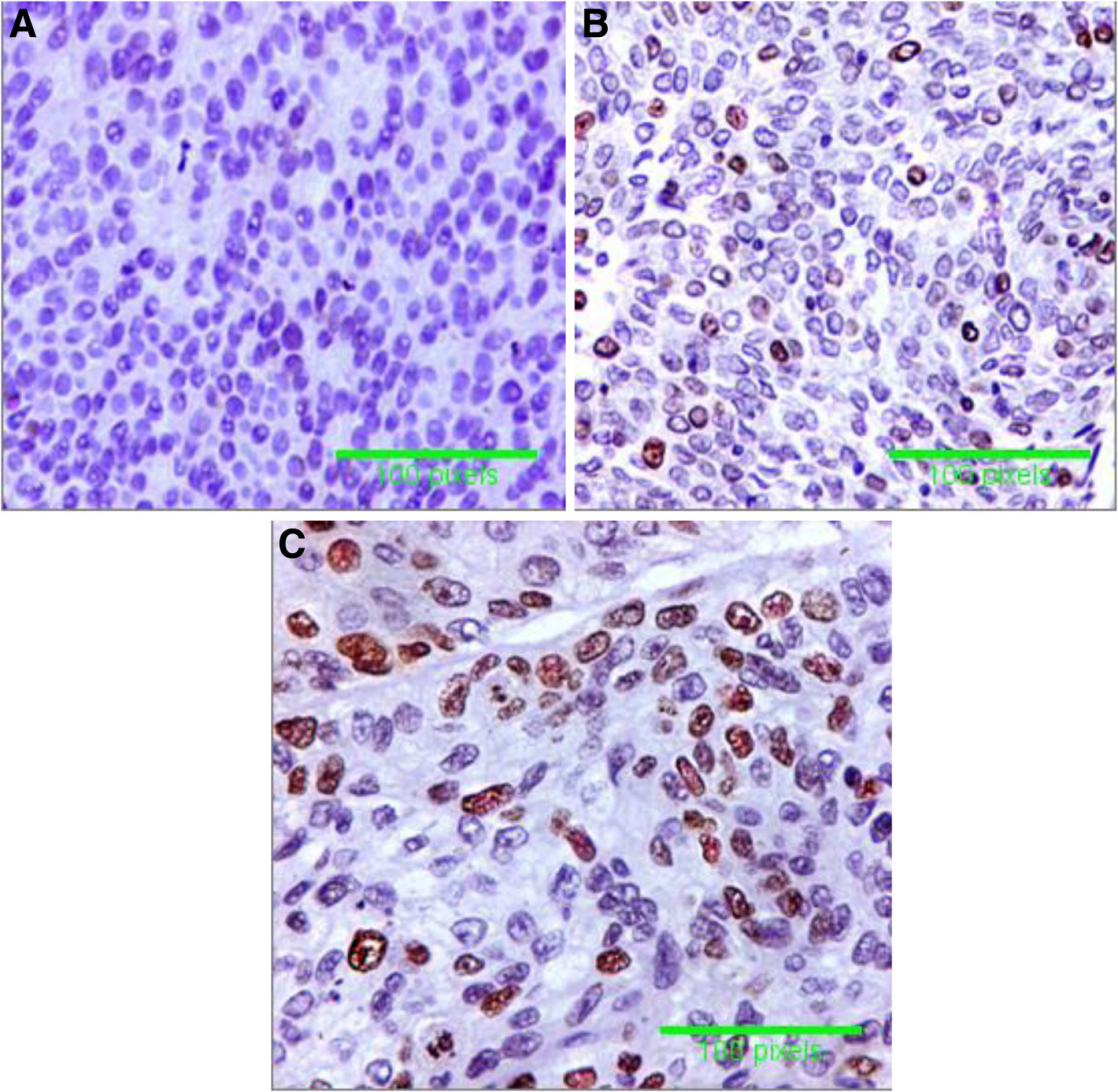
**Specimens of Transitional cell carcinoma (TCC). A** = TCC with negativity for telomerase (hTERT), **B** = TCC with intranuclear dot like positivity of hTERT in more than 25% of tumour cells from non-HCV infected patients, and **C** = TCC with intranuclear dot like positivity in more than 50% of tumour cells from HCV infected patients (immunoperoxidase DAB X400).

The immunoreactivity for hTERT in tissue sections was 6.0% (3/50) in normal urothelium of non-HCV infected patients (with mean labeled cells 11.53 ± 2.82), and 24% (12/50) of normal urothelium of HCV infected patients (with mean labeled cells 14.90 ± 1.46). Also, altered immunoreactivity for hTERT was 48% (24/50) in malignant urothelium of non-HCV infected patients (with mean labeled cells 31.65± 9.02) and 76% (38/50) in malignant urothelium of HCV infected patients (with mean labeled cells 44.36 ± 9.84) (p < 0.001) (Table 3). hTERT immunostaining appeared as intranuclear dot like positivity which was mainly localized in the nucleolus (Figure 1).

**Table 3:** Expression of studied genes in HCV-infected and non HCV-infected normal and malignant urothelium by real time PCR

	**Normal urothelium without HCV**	**Normal urothelium with HCV**	**Malignant urothelium without HCV**	**Malignant urothelium with HCV**
**Telomerase :**				
**• hTERT by RT-PCR**	0.13 ± 0.05	2.37 ± 0.44^a^	6.16 ± 0.57 ^ab^	11.88 ±1.34 ^abc^
**• hTERT by IH** (+ve cases)	3 (6%)	12 (24%)	24 (48%)	38 (76%)
Mean labeled cells	11.53 ± 2.82	14.90 ± 1.46	31.65 ± 9.02	44.36 ± 9.84
**TRAP (ng/ml)**	0.54 ± 0.24	2.98 ± 0.59 ^a^	8.03 ± 1.26 ^ab^	15.88 ± 1.94 ^abc^
(positive cases)	2 (4%)	10 (20%)	32 (64%)	41 (82)
**Tumour suppressor genes:**				
**• Rb by RT-PCR**	11.27 ± 1.42	6.92 ± 0.81 ^a^	2.75 ± 0.65 ^ab^	0.32 ± 0.30 ^abc^
**• Rb by IH (**positive cases**)**	3 (6%)	12 (24%)	26 (52%)	40 (80%)
**• E**_2_**F**_3_ by RT-PCR	0.41 ±0.09	1.74 ± 0.47 ^a^	3.60 ±1.22 ^ab^	9.75 ± 4.32 ^abc^
**• TP**_53_ by RT-PCR	0.25 ± 0.34	1.68 ± 0.29 ^a^	3.92 ± 0.46 ^ab^	12.39 ± 1.82 ^abc^
**• p53** by IH (positive cases)	0 (0%)	3 (6%)	21 (42%)	42 (84)
**• P**_21_ by RT-PCR	9.80 ± 0.45	5.83 ± 0.86 ^a^	3.19 ± 0.45 ^ab^	0.25 ±0.34 ^abc^
**• P**_21_ by **IH (**positive cases**)**	49 (98%)	31 (62%)	13 (26%)	3 (6%)
**Protooncogenes:**				
**• FGFR3 by RT-PCR**	0.08 ± 0.12	0.86 ± 0.76	3.06 ± 2.45 ^ab^	2.87 ± 3.88 ^ab^
**• FGFR3 by IH** (positive cases)	0 (0%)	2 (4%)	38 (76%)	48 (96%)

All data are expressed as Mean ± SD. One way ANOVA test with posthoc Scheffe’s test. ^a^ significant vs normal urothelium without HCV, ^b^ significant vs normal urothelium with HCV, ^C^ significant vs malignant urothelium without HCV (p< 0.05).

**Figure 2:**
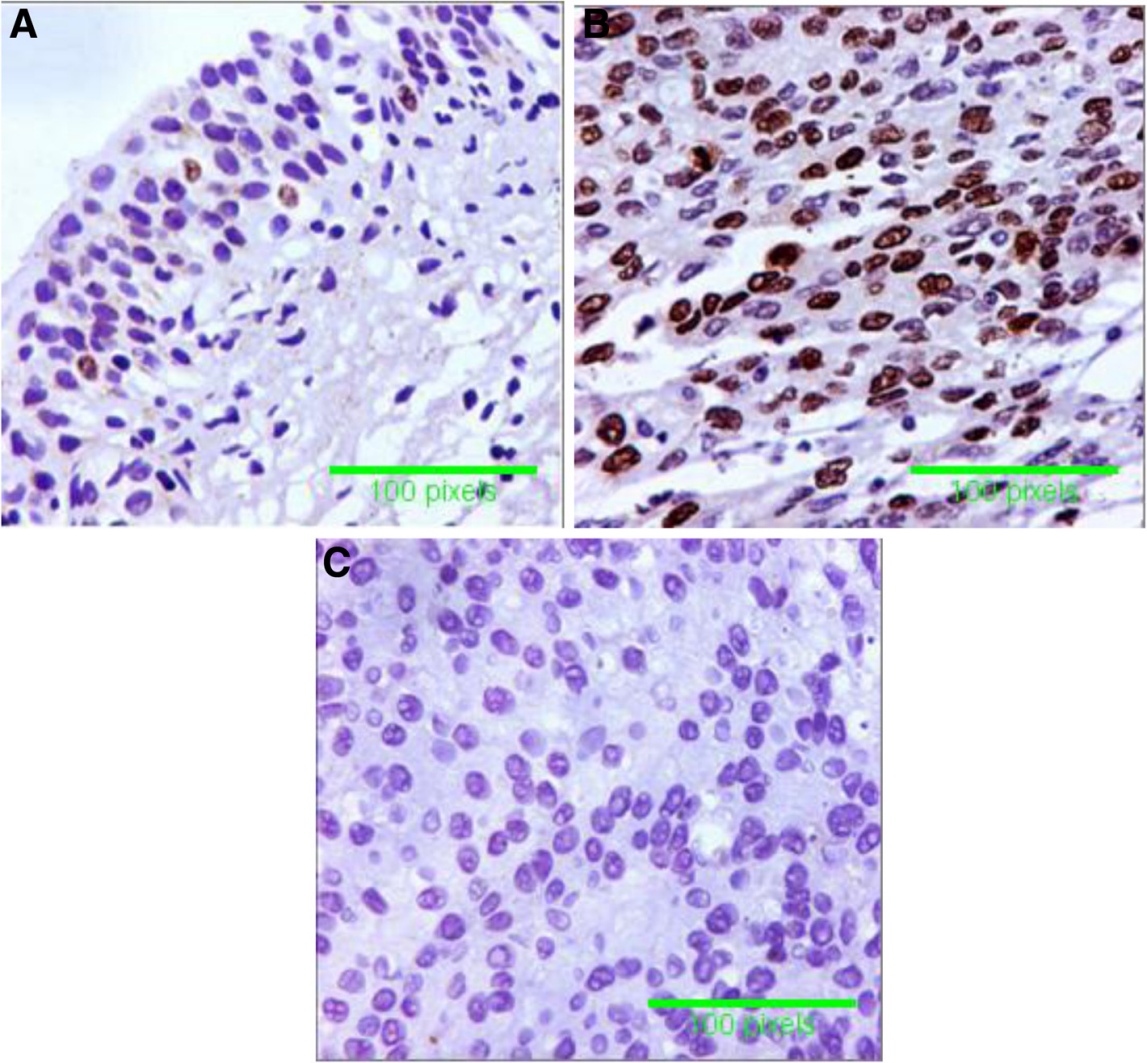
**Specimens of Bladder Cancer. A **= normal Rb heterogenous nuclear staining in less than 50% of tumor cells invasive carcinoma from non-HCV infected patients, **B **= altered Rb expression; homogenous strong staining for Rb in more than 50% of tumor cells from HCV infected patients and **C** = BC with negativity for Rb from HCV- infected patients (immunoperoxidase DAB X400).

The immunoreactivity for Rb showed that the altered expression for Rb protein was 6% (3/50) in normal urothelium of non-HCV infected patients, and was 24% (12/50) in normal urothelium of HCV infected patients. Also, altered immunoreactivity for Rb1 was 52 % (26/50) in malignant urothelium of non-HCV infected patients, and was 80% (40/50) in malignant urothelium of HCV infected patients (p< 0.001) (Table 3). Rb1 immunostaining appeared as strong homogenous nuclear staining in >50% of tumor cells (Figure 2).

**Figure 3:**
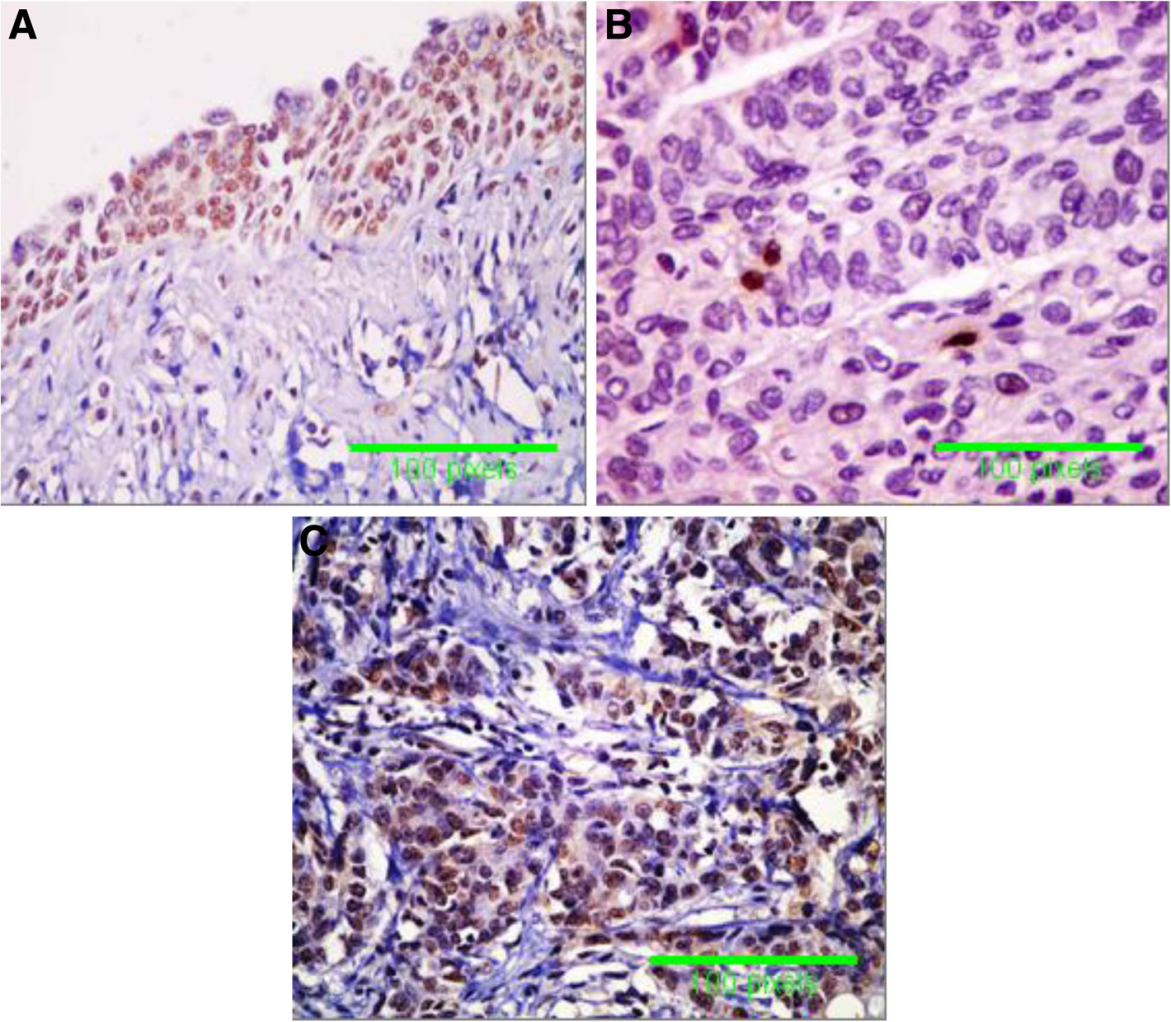
**Specimens of bladder urothelium. A **= non-neoplastic urothelium with nuclear immunoreactivity for p53 in more than 10% of urothelial cells, **B** = BC with nuclear immunoreactivity in less than 10% of tumor cells (scored negative) from non-HCV infected patients and **C** = BC with increased p53 expression in more than 10 of tumour cells in invasive carcinoma from HCV-infected patients (Immunoperoxidase DAB X 400).

**Figure 4:**
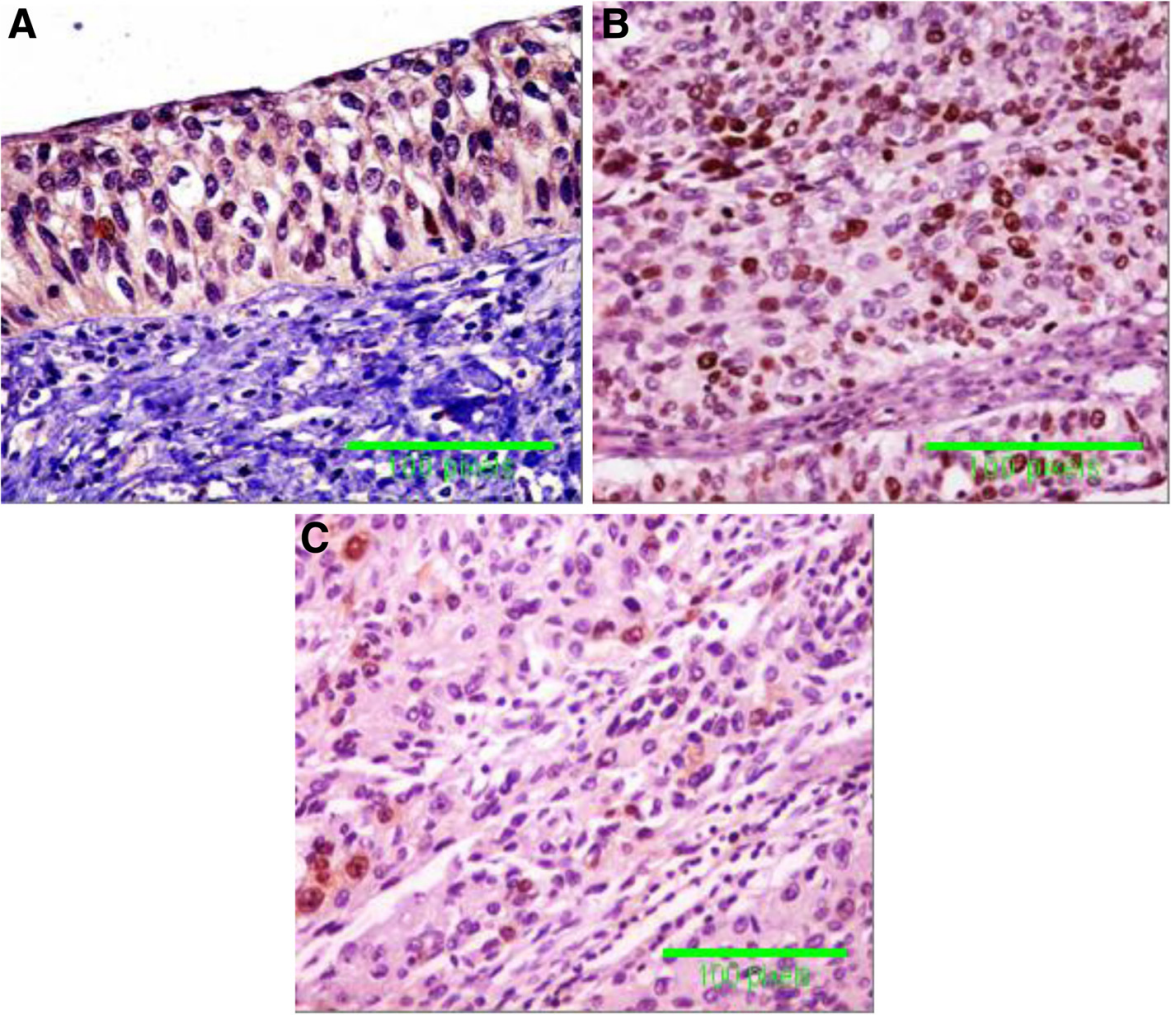
**Specimens of bladder urothelium. A **= non-neoplastic urothelium with no nuclear expression for p21 from HCV-infected patients, **B** = Transitional cell carcinoma (TCC) with nuclear immunoreactivity for p21 in more than 10% of tumor cells from non-HCV infected patients and **C** = lost p21 expression in most tumor cells from HCV infected patients (Immunoperoxidase DAB X 400).

**Figure 5:**
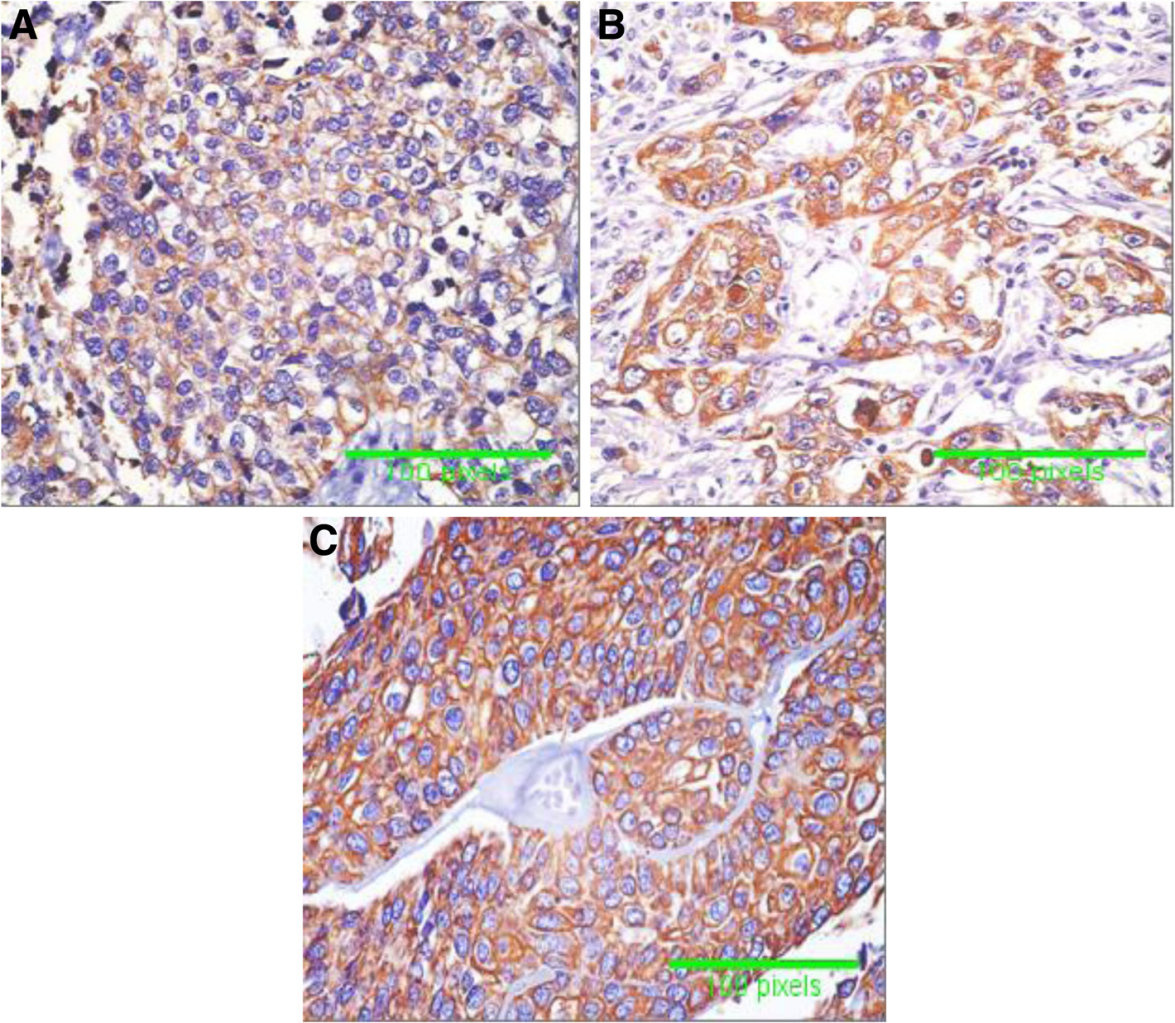
**Specimens of BC. A =** urothelial carcinoma with faint but detectable membranous and cytoplasmic positivity in tumor cells for FGFR3 from non-HCV infected patients, **B** = BC with moderate cytoplasmic positivity in most tumor cells for FGFR3 from HCV infected patients and **C** = BC with strong cytoplasmic positivity in tumor cells for FGFR3 from HCV infected patients (Immunoperoxidase DAB X400).

The immunoreactivity for TP53, CDKN1A (p21) and FGFR3 proteins was 0% (0/50) for TP53, 98% (49/50) for CDKN1A (p21), and 0% (0/50) for FGFR3 in normal urothelium of non-HCV infected patients and was 6% (3/50) for TP53, 62% (31/50) for CDKN1A (p21) and 4% (2/50) for FGFR3 in normal urothelium of HCV –infected patients (Table 3). The immunoreactivity was 42% (21/50) for TP53, 26% (13/50) for CDKN1A (p21) and 76% (38/50) for FGFR3 in malignant bladder tissues of non-infected patients, while the immunoreactivity was 84% (42/50) for TP53, 6% (3/50) for CDKN1A (p21) and 96% (48/50) for FGFR3 in bladder tumors associated of HCV-infected patients (p< 0.001). Immunostaining appeared as intranuclear positivity for TP53 (Figure 3), diffuse nuclear positivity for CDKN1A (p21) (Figure 4) and diffuse cytoplamic positivity for FGFR3 (Figure 5).

### Detection of telomerase activity by TRAP

In normal urothelium, TRAP activity was detected in 4% (2/50) of non-infected patients and 20% (10/50) of HCV-infected patients. But, TRAP activity in samples of malignant tissues was 64% (32/50) in non-infected patients, and 82% (41/50) in HCV-infected patients. Also, compared to normal urothelium of non-HCV infected patients, the activity of telomerase by TRAP was significantly high in normal urothelium of non-infected subjects (p< 0.001). In addition, TARP activity was significantly higher in malignant urothelium of HCV-infected subjects compared to malignant urothelium of non-infected subjects (Table 3).

### Detection of expression of hTERT, Rb, E2F3, TP53, CDKN1A (p21), and FGFR3 by real time RT-PCR

Quantitative real time PCR showed that the expression of these genes was higher in normal urothelium of HCV-infected patients compared to normal urothelium of non-infected patients (p< 0.001), except FGFR3 which showed non-significant change. In addition, the expression of such genes was significantly higher in malignant tissues of HCV infected patients compared to non-infected patients as well as compared to normal urothelium of HCV-infected and non-infected patients (p< 0.001) (Table 3).

On the other hand, Rb1, and CDKN1A (p21) gene expression was significantly higher in normal urothelium of non HCV infected patients compared to malignant tissues of non-HCV infected patients as well as compared to normal urothelium of HCV- infected and non-infected patients (p< 0.001) (Table 3).

### Correlations between HCV infection and expression of hTERT, Rb, E2F3, TP53, CDKN1A (P21), and FGFR3

There was a positive correlation between HCV infection and expression of hTERT, E2F3, TP53 and FGFR3 while Rb and CDKN1A (p21) showed negative correlation with HCV (p< 0.001) (Table 4).

**Table 4:** Correlations of HCV infection to expression of hTERT, Rb expression, E2F3, p53, p21 and FGFR3

	***r = correlation coefficient***	***p value***
1. hTERT expression vs. HCV infection	0.931	0.000
2. Rb expression vs. HCV infection	−0.901	0.000
3. E2F2 expression vs. HCV infection	0.427	0.000
4. p53 expression vs. HCV infection	0.730	0.000
5. p21 expression vs. HCV infection	−0.567	0.000
6. FGFR3 expression vs. HCV infection	0.871	0.000

### Survival analysis

**Figure 6:**
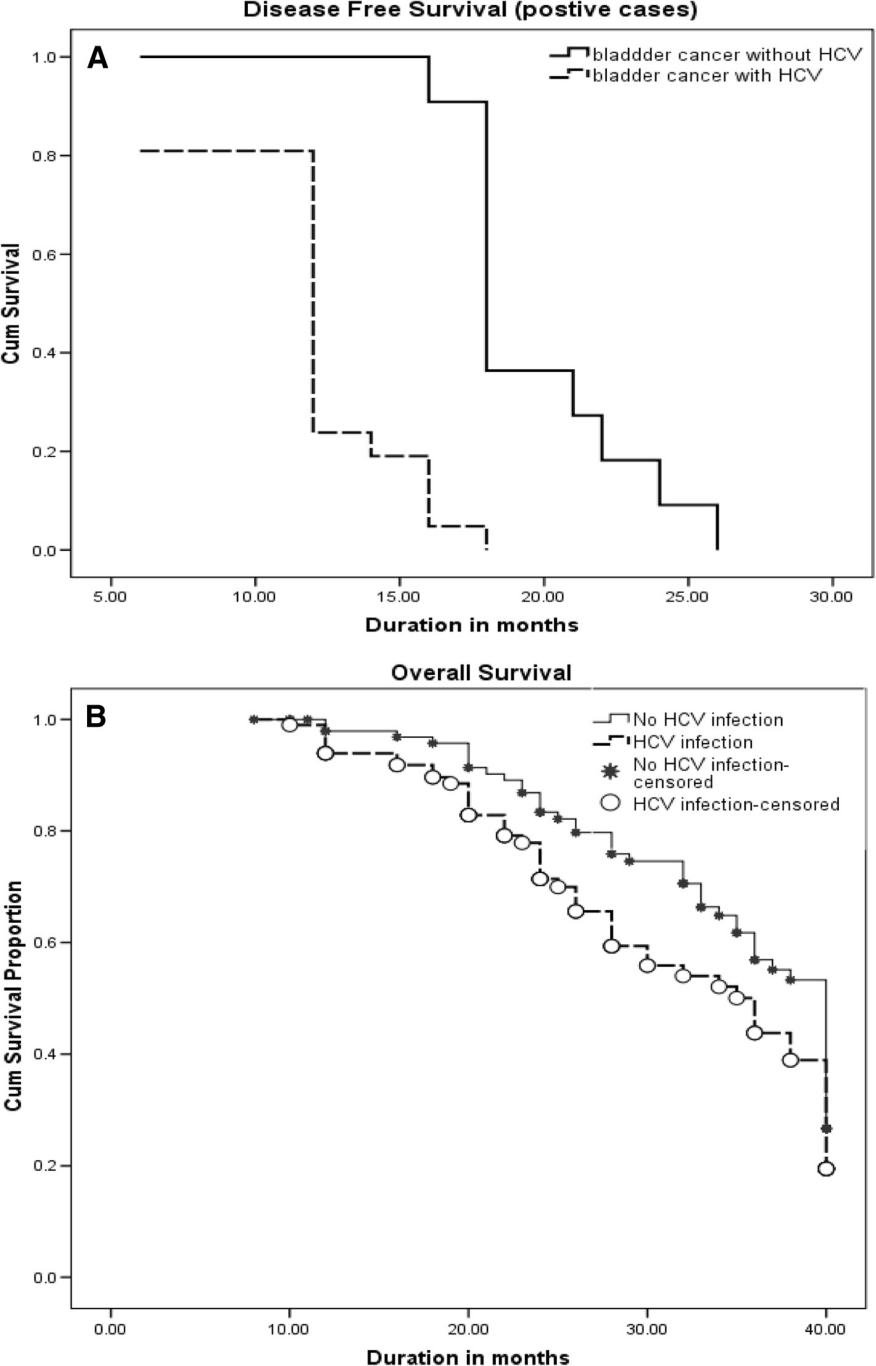
**Kaplan-Meier analysis for cases of BC.** Patients with HCV infections showed significantly lower overall survival rate (**A**), and disease free survival (positive case) (**B**).

The overall (OS) and disease free survival (DFS) curves showed a statistically significant decrease in overall survival and disease free survival in patients with HCV accompanied with bladder cancer (p = 0.047, p= 0.029 by Log-Rank test) (Figure 6).

## Discussion

In the context of the many associations between a virus and a given malignancy, the distinction between associated versus causative agent frequently arises and may be difficult to make [29]. The major pathogenetic role of HCV infection in hepatocellular carcinoma is well documented [18,19]. The mechanism of its oncogenesis remains unclear; however, alterations in cell cycle, proto-oncogenes, tumour suppressor genes, apoptotic proteins, telomeres are the key events in determining the biological behavior of bladder cancer [2,17,30]. In the present study we examined the expression of the telomerase and tumour suppressor genes (Rb, E2F3, TP53, and CDKN1A; p21), and proto-oncogenes (FGFR3) genes in HCV- and non-HCV infected patient with bladder cancer.

In the present study we found that, the BC cases associated with HCV infection were of TCC type, higher grade, and more invasive while, non-HCV-associated cancers were of SCC type, lower grade, and less invasive tumors. Moreover, the present study showed positive correlation between TCC and HCV infection, suggesting that the HCV infection might be a risk factor for bladder cancer of TCC cell type. On the other hand, *Abdulamir et al.,*[31] reported that schistosomal bladder tumors (SBT) were associated significantly with SCC, high grade, and invasive tumors while non-SBT were associated with TCC, a bit lower grade, and less invasive tumors.

*Yoshida* and *Toge*[32] hypothesized that telomerase might be activated in early stages of urological carcinogenesis. Expression of hTERT by real time PCR in the present study showed significant increase in normal urothelium of HCV infected patients as well as in malignant bladder tissues from HCV infected patients. Also, detection of hTERT by immunohistochemistry in tissue samples showed expression in 6% in normal urothelium from non- HCV infected patients and 24% in that from HCV infected patients. In malignant tissues, immunostaining was 48% in samples from non-HCV infected patients with mean labeled cells 31.65± 9.02 and 76% in samples from HCV-infected patients with mean labeled cells 44.36 ± 9.84. We found that hTERT is localized predominantly in the nucleolus and this is in agreement with the few previous reports describing hTERT protein localization [25,33] as the nucleolus is the site of nucleoprotein complex assembly [34]. These findings suggest that HCV infection enhances the expression of telomerase enzyme in normal and malignant tissues. Also, the activity of telomerase was evaluated by TRAP assay which is the most widely used method for monitoring telomerase activity. In this study, TRAP was positive in 4% of samples of normal urothelium from HCV-infected patients and 20% from HCV infected patients. In malignant tissues, TRAP was positive in 64% of non HCV infected patients and 82% of HCV infected patients. Detection of telomerase activity in normal urothelium of patients with BC suggests that telomerase might be activated in the early stages of BC carcinogenesis. Our findings are in agreement with *Yoshida* and *Toge*[32] who reported the activity of telomerase by TRAP in more than 70% of bladder cancer and *Abd El Gawad et al.*[35]. *and Abdel-Salam et al.*[36] who reported positivity of TRAP in 73.9% of cases with bilharzial cancer and 87% for non bilharzial BC. The absence of telomerase activity in some tumors may be due to the presence of a telomerase inhibitor [17]. Also, the presence of a positive correlation between HCV infection and TRAP and hTERT expression suggests that HCV infection might have a role in development and progression of BC especially of the TCC type.

Tumour suppressor genes are involved in the process of oncogenesis. We tested in our study retinoblastoma (Rb), and TP53 genes. It appears conceivable that TP53 may negatively regulate the expression of genes through the induction of *p21*/*CDKN1A* and the consecutive hypophosphorylation of pRb and its relatives [37]. Retinoblastoma tumor suppressor (Rb) gene encodes a nuclear phosphoprotein (pRb) that functions as a cell cycle regulator [5]. Unphosphorylated pRb negatively regulates E2F, a protein transcription factor, by binding with it. The transcription factors E2f1, E2f2 and E2f3 act as promoters of the G/S phase introduction, E2f4, E2f5, and E2f6 are generally regarded either as weak transcriptional activators or as transcriptional repressors [6]. This protein species, in turn, binds to E2F family transcription factors and converts them from transcriptional activators to transcriptional repressors [37]. When pRb is phosphorylated by the cyclin/CDK complex, the transcription factor E2F-1 is released and switches on genes (e.g. thymidine synthetase) whose products drive cells into the DNA synthesis (S) phase of the cell cycle. Normal cells express the Rb protein, while mutations or gene deletions, which often result in lack of protein expression, may be identified by the lack of Rb expression [6]. Previous studies found that Rb gene mutations are seen in approximately 30% of BC [5] and reported that co-operation between (pRb) removal and over expression of E2F3 may be required for bladder carcinogenesis [6]. In the present study, immunohistochemical detection of Rb protein in tissue samples of Rb expression showed significant increase in altered expression (either negative or increased homogenous positivity in >50% of cells) in bladder tumors associated with HCV infection. In addition, we found that Rb expression by RT-PCR was decreased in bladder tumors from HCV infected patients. As an inhibitor of cyclin-dependent kinases, p21 is known to prevent the phosphorylation of retinoblastoma (Rb) [38] family proteins and hence lead to the accumulation of hypophosphorylated pRb [39].

Also, we recorded overexpression of E2F3 in malignant BC when compared to normal urothelium, and this overexpression is enhanced by HCV infection. Moreover, Rb had a negative correlation with HCV infection, and positive correlation with TCC, while, it has no correlation with the grade and invasiveness of bladder cancer.

Functional inactivation of TP53 is the most common event in human malignancies, occurring in at least half of all tumors [40]. In the present study, immunohistochemical detection of TP53 in tissue samples from normal urothelium of non HCV infected patients revealed negative staining, while positivity was 6% of samples from normal urothelium from HCV-infected patients. Malignant tissues from non-HCV infected patients showed positivity in 42% of samples and those from HCV infected patients showed positivity in 84% of samples. In consistence with immunohistochemical findings, assay of TP53 by RT-PCR showed significant increase in its expression in normal urothelium from HCV-infected patients and in malignant tissues from HCV infected patients. Moreover, TP53 expression showed positive correlation with HCV infection. TP21 (CIP1/WAF1) cyclin kinase inhibitor protein; binds to and inhibits the activity of cyclin-CDK2 or -CDK1 complexes, and thus functions as a regulator of cell cycle progression at G_1_[41]. The expression of this gene is tightly controlled by the tumor suppressor protein TP53, through which this protein mediates the TP53-dependent cell cycle G_1_ phase arrest in response to a variety of stress stimuli. Although, CDKN1A (p21) is a transcriptional target of the tumor suppressor gene TP53, unfortunately, the present study, showed significant loss in CDKN1A expression in bladder tumors from HCV- infected patients. In addition, we found low CDKN1A expression by RT-PCR in bladder tumors from HCV infected patients. Moreover, CDKN1A had a negative correlation with HCV infection. However, we could not explain the cause for this controversy in decreased expression of CDKN1A in HCV infected patients in contrast to TP53. Decreased or loss of CDKN1A expression in BC of HCV infected patients could be a sign for bad prognosis in case of BC. In a previous study of patients with advanced bladder carcinoma undergoing radical surgery showed that patients with tumors that maintained CDKN1A (p21) expression had increased survival relative to patients with loss of CDKN1A expression [42].

The last tested gene is FGFR3 (proto-oncogene) which is associated with early papillary lesions with low malignant potential [9,10]. The mutations of FGFR3 are found more frequently in superficial than in invasive urothelial cell carcinoma (UCC) [9], and it has been reported that such mutations are more frequent in UCCs that do not recur [10]. Real time PCR and immunohistochemical examination of FGR3 in the present study showed high expression of FGFR3 in malignant bladder tissues from HCV infected patients. Moreover, there is a positive correlation between FGFR3 and HCV infection. Finally, an interesting and novel finding in the present study was a negative correlation between HCV infection and overall survival rates and disease free survival.

Although, the present study was the first study, up to the best of our knowledge, demonstrating altered expression of telomerase, Rb, E2F3, TP53, CDKN1A (p21) and FGFR3 in BC patient with HCV infection, it has some limitations. This study did not investigate mutations of these genes in BC of HCV infected patients. Moreover, the effect of HCV on chromosomal stability, and DNA repair genes, hence the behavior of bladder cancer, was not studied. Also, decreased overall survival and disease free survival rates of BC with HCV may be due to other factors such as liver affection, other metabolites, medications, and immune system alterations, and this point needs further exploration to study the impact of these factors on survival of HCV infected patients. This would be a future proposal for further study and correlations.

## Conclusion

HCV infection is associated with TCC of higher grade and more invasiveness. The expression of hTERT, Rb, E2F3, TP53 and FGR3 as well as the activity of telomerase were significantly high in malignant bladder tissues associated with HCV infection. On the other hand, CDKN1A (p21) expression was significantly low in BC associated with HCV infection. Moreover, there were positive correlations between HCV infection and expressions of telomerase, E2F3, TP53 and FGFR3 expression and negative correlations between HCV infection and expression of Rb and CDKN1A (p21) genes. Further studies are recommended to investigate the possible role of HCV infection in pathogenesis of bladder cancer. Furthermore, whenever the risk factors are demonstrated using these tools, it might dictate an additional new adjuvant therapy with surgery to improve the survival of patients at high risk. Furthermore, easily detection and treatment of active HCV infections is warranted.

## Competing interests

The authors declare that they have no competing interests.

## Authors’ contributions

**KZH** contributed to data collection and study design. **AA** carried out molecular study of investigated genes by real time PCR. **EE** carried out molecular study for HCV infection. **A-aMH** is the corresponding author and participated in data collection and analysis and manuscript drafting. **AAS** collected collection of tumour samples and participated in manuscript drafting. **AA-a** carried out histopathological assessment routine and immunostaining. **ASE-H** collected collection of tumour samples. **HA-e** contributed to putting the hypothesis and study design and collection of tumour samples. All authors read and approved the final manuscript.
